# Current Understanding of Temperature Stress-Responsive Chloroplast FtsH Metalloproteases

**DOI:** 10.3390/ijms222212106

**Published:** 2021-11-09

**Authors:** Shengji Luo, Chanhong Kim

**Affiliations:** 1 Shanghai Center for Plant Stress Biology, CAS Center for Excellence in Molecular Plant Sciences, Chinese Academy of Sciences, Shanghai 200032, China; shjluo@psc.ac.cn; 2 University of the Chinese Academy of Sciences, Beijing 100049, China

**Keywords:** FILAMENTOUS TEMPERATURE-SENSITIVE H, protein quality control, proteostasis, retrograde signaling, reactive oxygen species

## Abstract

Low and high temperatures are life-threatening stress factors, diminishing plant productivity. One of the earliest responses of plants to stress is a rapid burst of reactive oxygen species (ROS) in chloroplasts. Widespread efforts over the past decade shed new light on the chloroplast as an environmental sensor, translating the environmental fluctuation into varying physiological responses by utilizing distinct retrograde (chloroplast-to-nucleus) signals. Recent studies have unveiled that chloroplasts mediate a similar unfolded/misfolded/damaged protein response (cpUPR) as observed in the endoplasmic reticulum and mitochondria. Although observing cpUPR is not surprising since the chloroplast is a prime organelle producing harmful ROS, the intertwined relationship among ROS, protein damage, and chloroplast protein quality controls (cpPQCs) with retrograde signaling has recently been reported. This finding also gives rise to critical attention on chloroplast proteins involved in cpPQCs, ROS detoxifiers, transcription/translation, import of precursor proteins, and assembly/maturation, the deficiency of which compromises chloroplast protein homeostasis (proteostasis). Any perturbation in the protein may require readjustment of proteostasis by transmitting retrograde signal(s) to the nucleus, whose genome encodes most of the chloroplast proteins involved in proteostasis. This review focuses on recent findings on cpUPR and chloroplast-targeted FILAMENTOUS TEMPERATURE-SENSITIVE H proteases involved in cpPQC and retrograde signaling and their impacts on plant responses to temperature stress.

## 1. Introduction

The climate crisis, driven mainly by escalating CO_2_ emissions (https://www.ipcc.ch (accessed on 1 October 2021)), exacerbates global warming, accompanied by extreme and unpredictable weather changes worldwide. This problem urges plant scientists to unveil mechanisms underlying plant thermotolerance and water stress to produce more sustainable crop plants toward climate changes and diminishing arable land. Accordingly, over the past several years, we observed an increasing number of research articles related to climate change, especially with great emphasis on thermotolerance, carbon sequestration, and unveiling the climate-adaptive genetic loci using a set of geographically diverse natural variants [1,2,3,4].

Exploring plant basal and acquired thermotolerance has enabled the finding of vital molecular components, which play decisive roles in heat stress (HS) responses [5,6]. The basal response is determined by an inherent aptitude of plants toward the level of optimal growth temperature. In contrast, an acquired thermotolerance can be practically gained against lethal HS (i.e., preacclimation by mild HS prior to lethal HS). Nonetheless, the shared consequence of these seemingly different HS is the accumulation of unfolded/misfolded/damaged proteins, which to a certain level can be controlled through a process called unfolded protein response (UPR) [6]. Endoplasmic reticulum and mitochondrial UPR (erUPR and mtUPR, respectively) have been reported to play essential roles for protein-folding homeostasis under oxidation-prone stress conditions [7,8,9]. Although chloroplasts overproduce harmful reactive oxygen species (ROS) upon exposure to various oxidative stress conditions, including heat and cold stresses, whereby Rubisco alone accounts for over 60% of total foliar soluble proteins [10], the chloroplast UPR (cpUPR) has recently been identified in mutants of *Arabidopsis thaliana* (*Arabidopsis*) and *Chlamydomonas* defective in chloroplast proteostasis [11,12,13]. Like heat stress, cold stress also leads to ROS accumulation in chloroplasts [14,15]. However, it is unclear whether cpUPR also contributes to plant cold stress response(s). It is also important to note that chloroplasts can detoxify harmful ROS through enzymatic and nonenzymatic systems [16]. However, under long-lasting or severe stress conditions, the level of ROS exceeds the scavenging capacity of chloroplasts, concurrently enabling activation of signaling and oxidative damage [17].

In the eukaryotic protein quality control (PQC) system, some chaperones refold unfolded/misfolded proteins, while others facilitate their degradation through the 26S proteasome in the cytosol and nucleus. The post-translational modifications (PTMs), including ubiquitination, play a vital role in protein turnover. As endosymbionts, mitochondria and chloroplasts also utilize nuclear-encoded chloroplast chaperones and bacterial-type proteases for protein refolding and turnover [9,11,18]. Given that chloroplasts continually produce ROS, which alters protein structure and functionality, the correlated response of the nuclear genome to chloroplast PQC (cpPQC) status might be crucial for maintaining proteostasis under various ROS-overproducing stress conditions. This notion implies that, in addition to PTMs, mitochondrial and plastid proteostasis requires nuclear genome coordination to express a suite of genes needed for proteostasis in the targeted subcellular organelles.

Here, we mainly discuss topics regarding the multifaceted regulation of chloroplast proteostasis by explicitly focusing on the FILAMENTOUS TEMPERATURE-SENSITIVE H (FtsH) family in *Arabidopsis* [19], which paves the way for understanding chloroplast-mediated plant resilience toward a changing environment.

## 2. Chloroplast ROS-Mediated Operational Retrograde Signaling

Chloroplast ROS, byproducts of photosynthesis, are accountable for the photodamage of chloroplast macromolecules, such as proteins, lipids, and nucleic acids [20,21,22,23,24]. These harmful ROS produced in chloroplasts include singlet oxygen (^1^O_2_), superoxide anion (O_2_^−^), hydrogen peroxide (H_2_O_2_), and hydroxyl radical (HO•) [16,25,26]. Since the various foliar metabolisms significantly rely on chloroplasts, it is indisputable that the ROS-driven chloroplast photodamage will negatively affect plant productivity, especially under fluctuating, severe, and long-lasting stress conditions. However, independent studies have revealed that chloroplast ROS can be beneficial for plants to adapt to distinct stress factors by triggering various signaling pathways from the stressed chloroplasts back to the nucleus, collectively referred to as operational retrograde signaling (ORS) [22,27,28]. For instance, the ROS-driven oxidation of proteins and an antioxidant compound (β-carotene) were found to be integral for activating ORS pathways in response to external stimuli, such as high light, cold, drought, and herbivores, thereby linking chloroplast photodamage to signaling [23,29,30,31]. Like PTMs, including phosphorylation, acetylation, ubiquitination, and SUMOylation, critical for modulating stress signaling, ROS-driven oxidative PTMs and subsequent conformational changes and/or turnover seem to modulate retrograde signaling to coordinate cellular stress responses.

## 3. Chloroplast FtsH-Dependent Proteostasis Functions in Photoprotection

Among those chloroplast proteases implicated in cpPQC, the ATP-dependent zinc metalloprotease FtsH family is involved in plant resilience against heat and cold stress. FtsHs are integral membrane proteases comprising an AAA (ATPase associated with various cellular activities) and a Zn^2+^ protease domain, universally found in eubacteria, chloroplasts, and mitochondria [32,33,34]. Typically, FtsH proteases are barrel-shaped homo or hetero hexamers anchored in the membrane through one or two transmembrane domains. FtsH proteases unfold protein substrates through AAA-dependent ATP hydrolysis and degrade them in a proteolysis chamber. The bacterial genome harbors only one single FtsH, critical to confer high temperature-tolerant division [35], except for the cyanobacterial genome with four FtsH genes [36,37]. The oxygenic photosynthesis most likely resulted in the multiplication of FtsH in cyanobacteria [38,39]. Intriguingly, a recent study demonstrated that FtsH also regulates bacterial transcription by proteolytically modulating the activity of an atypical membrane-bound transcription factor in the Gram-positive pathogen *Staphylococcus aureus* [40]. In *Escherichia coli*, FtsH also participates in membrane lipid biosynthesis by proteolytically regulating the level of LpxC, a deacetylase catalyzing the first step of lipid A biosynthesis, thereby sustaining the membrane permeability barrier [41,42]. Yeast contains three FtsH genes whose functions are implicated in thermotolerance, protein import, and proteostasis [43]. In contrast to prokaryotes and yeast, there are 12 nuclear-encoded FtsH proteases in *Arabidopsis*, among which nine FtsHs function in chloroplasts and three FtsHs in mitochondria [44]. It became increasingly clear that FtsH proteases are essential for sustaining membrane PQC, alleviating cold- and heat-induced chloroplast damage [24,44,45,46].

In plants, the thylakoid membrane-anchored hetero-hexameric FtsH complex has been widely studied due to its involvement in chloroplast biogenesis and PSII quality control [47,48]. Four isomers, i.e., type A FtsH1 and FtsH5, as well as type B FtsH2 and FtsH8, comprise the hetero hexamer assembled in the non-appressed regions, such as the grana margin and stromal lamellae [49,50]. Nonetheless, given the striking foliar phenotype (so-called leaf variegation) of *var2* (*ftsh2*) and *var1* (*ftsh5*) mutants and higher protein abundance among their isomers, FtsH2 and FtsH5 are considered significant subunits of the hetero hexamer. In addition to the leaf variegation, *var2* and *var1* mutants exhibit extreme sensitivity toward high-light and cold stresses because of the failure of PSII quality control [24,48].

Chloroplasts overproduce ROS through PSII and PSI under various stress conditions, such as cold, heat, and high light [16]. Among those ROS, PSII-generated ^1^O_2_ has been widely accepted as a major ROS in damaging PSII core proteins, including D1. The ROS-damaged PSII core proteins undergo proteolysis via PSII quality control in the PSII repair cycle [47,51]. For instance, the partially disassembled PSII core complex exposes the damaged D1 protein, enabling its proteolysis by the thylakoid membrane-bound hetero-hexameric FtsH protease. Hence, the loss of either FtsH2 or FtsH5 would increase the amount of ^1^O_2_-damaged PSII core proteins [23,24,52]. The accumulation of damaged PSII core proteins triggers cpUPR in the *var2-9* mutant harboring a missense mutation in the AAA domain of FtsH2, impairing substrate-unfolding activity [18]. The *var2-9*-induced cpUPR genes involved in cpPQC include genes encoding chaperones, proteases, and ROS detoxifiers. A similar cpUPR was also observed in *Arabidopsis* and *Chlamydomonas* mutants with reduced stromal Clp protease activity [11,12], suggesting that a defect in either membrane proteostasis or stromal proteostasis can trigger cpUPR through a yet unknown signaling mechanism. However, since Clp protease is known to recognize both OXYGEN-EVOLVING ENHANCER PROTEIN 1-1 (PsbO) and polypeptides of the cytochrome *b_6_f* complex in the electron transfer chain, perhaps *clp*-induced cpUPR has partly resulted from defective membrane proteostasis [53,54]. Nevertheless, both *var2-9* and *clp* mutants exhibit higher expression of *HEAT-STRESS TRANSCRIPTION FACTOR A-2* (*HSFA2*) than wild-type plants. HSFA2 then drives the expression of various chaperones, such as heat-shock proteins (HSPs). In particular, the increased levels of chloroplast-localized HSP70 and ClpB3 chaperones seem to be essential for refolding the aggregated enzyme in the plastidial methylerythritol phosphate (MEP) pathway under oxidative stress conditions [11,18,55], suggesting that the MEP pathway tends to be deactivated in *var2-9* and *clp* mutant plants. Since the MEP pathway is essential for maintaining photosynthesis and directly linked to the activation of retrograde signaling, a deactivation of the MEP pathway and/or accumulation of certain intermediates may trigger appropriate adaptive stress responses [27,56]. Indeed, one of the intermediates, methylerythritol cyclodiphosphate (MEcPP), induces a suite of nuclear genes encoding the core erUPR proteins [57], suggesting that chloroplast retrograde signals can modulate ER stress. However, it is unclear whether MEcPP can also induce cpUPR.

Moreover, the defective membrane proteostasis in the *var2-9* mutant increases cellular salicylic acid (SA) content via the chloroplast isochorismate (ICS) pathway without transcriptional reprogramming of nuclear genes involved in the ICS pathway such as *ISOCHORISMATE SYNTHASE 1* (*ICS1*) [24]. Similar to *var2-9*, the *var2* null mutant is found to rapidly induce SA-responsive genes upon exposure to combined high-light and cold stress (PSII-damaging stress conditions). Hence, it is conceivable that the defective proteostasis and accumulation of damaged proteins, such as PSII core proteins, in both *var2-9* and *var2* null mutants may simultaneously stimulate the ICS pathway. Moreover, an uncoupled accumulation of SA to the *ICS1* transcript abundance implies that the rapidly accumulated SA through a post-translational activation of the chloroplast ICS pathway may directly mediate retrograde signaling in *var2* mutants. Examining the expression levels of cpPQC genes in *var2* mutants with depleted SA (e.g., by expressing bacterial SA-hydrolyzing enzyme NahG) may also prove whether SA is involved in the expression of cpPQC genes to reduce chloroplast photodamage. Notably, a high cellular SA level modulates ER stress and UPR [58], indicating that SA and MEcPP signaling are intertwined with the erUPR.

Like the PSII reaction center, the light-harvesting antenna complex of PSII (LHCII) also inevitably produces ROS in the process of energy capture, damaging LHCII proteins [17,25]. While the thylakoid membrane hetero-hexameric FtsH protease removes the damaged PSII core protein D1, the LHCII turnover mechanism is relatively unclear. Despite the uncertainty, among those LHCII proteins, Lhcb1 and Lhcb3 were previously shown to undergo FtsH6 protease-dependent proteostasis under high-light stress and during dark-induced senescence, respectively [59]. However, whether both LHCII proteins are genuine substrates of FtsH6 or FtsH6 mediates their proteolysis through other membrane proteases was not addressed. Likewise, it is unknown if FtsH6 functions as a homo- or hetero-hexameric complex in the thylakoid membrane. Moreover, the prevailed post-translational modification of LHCII proteins, i.e., phosphorylation, under fluctuating light conditions was not associated with their turnover [60]. Perhaps, the ROS-dependent oxidative modification may stimulate their turnover through an oxidation-driven conformational change. As for D1, the N-terminal domain of LHCII is critical for its acclimation-prone proteolysis [61]. Thus, if the ROS-dependent conformational change of LHCII triggers its proteolysis, the structural change may occur in the N-terminal region. In contrast to the proposed function of FtsH6 toward photo-acclimative LHCII turnover, the Arabidopsis mutant lacking FtsH6 behaves like wild-type plants under photoinhibitory conditions [37]. Under the same experimental conditions, not *ftsh6* but only *var1* and *var2* mutants showed a drastic decline in PSII activity, raising the question of the biological relevance of LHCII turnover by FtsH6. Consistently with this notion, FtsH6 was found to be dispensable for LHCII protein degradation in planta [44].

## 4. Thylakoid Membrane Ftsh6 and Hsp21 Modules Function in Thermomemory

After more than 10 years from the initial report on FtsH6 function, Sedaghatmehr et al. [62] demonstrated that FtsH6 plays a negative role in sustaining thermomemory by promoting HEAT-SHOCK PROTEIN 21 (HSP21) turnover in chloroplasts. It was suggested that maintaining acquired thermotolerance relies on upholding high levels of chloroplast-targeted HSP21 during the HS recovery phase (several days without external stimuli), referred to as thermomemory [62]. This maintained thermomemory with retained high levels of HSP21 confers thermotolerance to plants subsequently treated with lethal HS. Hence, the *Arabidopsis ftsh6* mutant exhibits enhanced thermomemory and thermotolerance to lethal HS. However, whether HSP21 is a direct substrate of FtsH6 protease or whether FtsH6 drives HSP21 turnover by forming a hetero hexamer with other FtsH proteases remains unknown. Alternatively, FtsH6 may facilitate HSP21 degradation through distinct chloroplast proteases. The same research group later revealed that not only FstH6 but also autophagy (implicated in both selective degradations of plastid proteins and plastids) is required for HSP21 degradation in the vacuole [63]. Consistently, inactivation of both FtsH6 and autophagy greatly improves thermomemory with an increased level of HSP21 during the HS recovery. In sharp contrast to the thermomemory-driven thermotolerance, it was shown that the responses regarding basal and acquired heat-stress tolerance in *ftsh6* mutant plants are comparable to those in wild-type control plants. Since heat stress rapidly induces both FtsH6 and its likely substrate HSP21 [64], it is puzzling why the *ftsh6* mutant shows comparable basal and acquired thermotolerance levels relative to wild-type plants. Perhaps, other chaperone-protease modules may function for either the basal and/or the acquired thermotolerance. It is also unknown whether the lack of FtsH6 can continually induce cpUPR as shown in other chloroplast protease mutants, such as *var2* and *clp*. On the other hand, it would be interesting to examine *var2* and *clp* mutant plants in the context of their thermomemory and HS responses since both mutants constitutively express the HSF2A transcription factor accountable for *HSP21* expression [11,18,65,66].

## 5. Chloroplast Envelope FtsH11 Mediates Thermotolerance

Another FtsH protease, FtsH11, has been linked to achieving thermotolerance in *Arabidopsis* [45,46,67]. Even though Ftsh11 is exclusively located in the chloroplast envelope membrane, moderate heat stress notably compromises photosynthetic activity (such as PSII quantum yield) in *ftsh11* mutant plants, mirroring the phenotypes of *var1* and *var2* mutants under high-light or cold stress. However, the heat-induced defective photosynthetic phenotypes of *ftsh11* were not observed under high-light stress conditions, where *var1* and *var2* mutants became susceptible owing to the failure of PSII repair [67], indicating a heat stress-specific activity of FtsH11 in chloroplasts. It seems that the impaired FtsH11-dependent proteostasis consequently impairs photosynthesis and promotes PSII turnover under moderate heat stress. Despite the apparent heat-sensitive phenotype, the related substrates of FtsH11 and how FtsH11 contributes to plant thermotolerance remain yet poorly investigated.

FtsH11 was first identified in *Arabidopsis* mitochondria, forming a heterocomplex with FtsH4, a homolog to the intermembrane (i)-AAA protease in yeast mitochondria [68]. However, the N-terminal signal sequence of FtsH11 targeted the fused GFP protein only into chloroplasts without any visible GFP signal in mitochondria [37]. Consistently, FtsH11 was detected in the chloroplast envelope fraction subjected to the mass spectrometry (MS) and immunoblot analyses [69,70]. Moreover, the latter study could not detect FtsH11 from purified mitochondria [70]. Although Adam and coworkers [46] detected mitochondria FtsH11 in isolated mitochondria from transgenic plants expressing HA-tagged FtsH11, the signal was conceivably weaker than in chloroplasts. Hence, the localization of FtsH11 needs to be further verified. Furthermore, the topology of FtsH11 in chloroplasts is yet unknown. Whether the ATPase and proteolytic domains face the stroma, the intermembrane space, or the cytosol needs to be confirmed to understand the mode of action of FtsH11 under HS.

Yeast mitochondrial i-AAA protease YME1, an *Arabidopsis* FtsH11 homolog, is anchored in the inner membrane with the AAA and proteolytic domains protruding into the intermembrane space (IMS) [71]. YME1 forms a homo hexamer comprising an ATPase ring to extract target substrates from the membrane and a proteolytic chamber to degrade unfolded substrates into small peptides (around 6–20 amino-acid residues) [9,72]. Multiple lines of evidence suggest that YME1 functions for nuclear-encoded mitochondrial protein import and inner membrane proteostasis. YME1 retrogradely translocates polynucleotide phosphorylase (PNPase) from the inner membrane import channel into the IMS [73]. The AAA-dependent chaperone activity rather than protease function seems to play a dominant role in translocating PNPase into the IMS. This finding implies that YME1 is required for mitochondrial protein import as the noncanonical import machinery, in addition to its known function in mitochondrial membrane protein turnover. If chloroplast FtsH11 shares a similar topology to yeast YME1, IMS proteostasis and/or IMS protein import can be potentially linked to the thermo-sensitive phenotype of *ftsh11*. By contrast, stromal proteins or peripheral thylakoid proteins facing the stroma can be prime targets of FtsH11 if the AAA and protease domains face the stroma. Recently, Adam et al. [46] suggested the chloroplast inner envelope membrane (IEM) TIC40 translocon protein as a potential substrate of FtsH11 because of its significant accumulation following HS. TIC40 is part of the motor complex associated with the import channel TIC110, stimulating the release of bound transit peptides from TIC110 and an ATP hydrolysis by another chaperone (HSP93) in the motor complex [74,75]. Additionally, affinity purification coupled with MS analysis demonstrated that FtsH11 interacts with CPN60 chaperonin involved in completing protein import and protein assembly in the stroma [46]. However, these results cannot fully determine the topology of FtsH11 since the level of CPN60 proteins was examined neither under nor after heat stress.

## 6. Protein Import-Associated Proteostasis Is Allied with Retrograde Signaling

The ubiquitin-proteasome system (UPS) degrades mitochondrial outer membrane (MOM) proteins [9]. The cytosolic AAA ATPase CDC48 is known to retrogradely extract ubiquitinated MOM proteins for UPS-mediated turnover, a process called the mitochondria-associated degradation (MAD) pathway. This process is similar to ERAD (ER-associated protein degradation) system in ER protein quality control [76]. Later, an MAD-independent pathway for MOM protein turnover was unveiled in yeast. Two YME1 adaptor proteins, Mgr3 and Mgr1, interact with MOM substrates through their IMS domains and directly recruit them to the YME1 complex to promote their degradation in IMS [77]. These MOM proteins include translocase of the outer mitochondrial membrane 22 (Tom22) and the outer membrane protein 45 (Om45) [78]. YME1 also interacts with substrates through its AAA and protease domains [79]. These findings demonstrate that MOM proteins undergo turnover in both locations, i.e., cytosol and IMS. MAD depends on eukaryotic UPS, whereas the MOM protein turnover in IMS requires YME1 originated from endosymbiotic α-proteobacteria.

Like mitochondrial MAD, chloroplast outer envelope membrane (OEM) proteins undergo proteolysis via a process named CHLORAD (chloroplast-associated protein degradation) [80,81,82]. In the process, the OEM-localized E3 ubiquitin ligase, named SP1 (suppressor of *ppi1*), ubiquitinates OEM translocon (TOC) proteins, promoting their degradation via the process coordinated by SP2 (OMP85-type beta-barrel), cytosolic CDC48, and the 26S proteasome. SP2 functions in the retrotranslocation of target proteins (from OEM to cytosol) using energy provided by the AAA ATPase CDC48, largely resembling the process of mitochondrial MAD [9]. Remarkably, under oxidative stress conditions, TOC proteins undergo a CHLORAD-mediated turnover to reduce chloroplast ROS levels, thereby compromising the import of photosynthesis-associated proteins [83]. These findings suggest that CHLORAD can constantly modulate the steady-state levels of chloroplast translocons under changing environmental conditions. However, the underlying mechanism via which the CHLORAD system is activated or deactivated remains to be elucidated. Alternatively, cytosolic or chloroplastic stress may lead to conformational changes in TOC proteins, enabling CHLORAD-mediated their turnover.

GUN1 (genomes uncoupled 1), a master integrator of biogenic retrograde signaling primarily implicated in chloroplast biogenesis, plays a critical role in chloroplast proteostasis through the interaction with transcription, RNA-editing, protein translation machinery components and import-aiding stromal chaperone proteins [11,27,84,85]. The import defect appears to result in the accumulation of nonimported chloroplast proteins in the cytosol. Then, cytosolic chaperone proteins, such as HP90 and HSP70, modulate the expression of nuclear genes (such as PhANGs) through yet unknown nuclear components, connecting chloroplast and cytosolic proteostasis for the activation of retrograde signaling [84]. At present, it is unclear whether CHLORAD-mediated TOC turnover participates in GUN1-mediated retrograde signaling. However, as shown in Wu et al. [84], it is plausible that TOC turnover leads to the accumulation of the nuclear-encoded chloroplast proteins in the cytosol, which may also trigger retrograde signaling through cytosolic HSP90 and HSP70 in a GUN1-independent manner.

## 7. Concluding Remarks

With climate change, temperature stress has become a more and more important matter for plant scientists. The short- and long-term heatwaves are challenging to endure for plants because of their sessile nature. Therefore, elucidating the underlying mechanisms of plant thermotolerance and thermomemory may provide a pivotal molecular toolbox for engineering HS-resilient crop plants. Indeed, many key components for priming plant thermotolerance have been revealed over several decades of research. However, although HS leads to chloroplast ROS burst and chloroplasts can act as environmental sensors, the role of chloroplast signaling in plant thermotolerance remains largely unexplored, especially for protein damage, quality control, and signaling. In this regard, this review mainly discussed the integral membrane FtsH proteases that play a pivotal role in cpPQC under temperature-related stress conditions. While thylakoid membrane-bound FtsH6 and FtsH1–FtsH2–FtsH5–FtsH8 protease complexes function in thermomemory and cold stress, respectively, the envelope membrane FtsH11 protease primarily contributes to thermotolerance. However, despite the apparent phenotypes of cognate mutants under related stress, many uncertainties remain to be elucidated, which include (i) FtsH6 topology and its associated protease(s), (ii) the role of FtsH1–FtsH2–FtsH5–FtsH8 protease complex under basal, acquired, and thermomemory-associated HS conditions, (iii) the precise location of FtsH11 in the chloroplast envelope and its topology, and (iv) the role of FtsH11 in protein import and cpPQC. A challenge for the future will be to resolve how cpPQC-linked retrograde signaling pathways are integrated into plant thermotolerance and thermomemory to confer robust physiological responses toward HS.
